# Nurses’ perceptions of the transition to 100% single-occupancy patient rooms in a university hospital in the Netherlands: an uncontrolled before and after study

**DOI:** 10.1186/s12912-024-01758-7

**Published:** 2024-02-08

**Authors:** Ralph Pruijsten, Erwin Ista, Jill Maben, Liesbeth van Heel, Monique van Dijk

**Affiliations:** 1 Section Nursing Science, Department of Internal Medicine, Erasmus University Medical Center, Rotterdam, the Netherlands; 2 School of Health Sciences, University of Surrey, Guildford, UK; 3 Department of Public Health, Real Estate Department, Erasmus University Medical Center, Rotterdam, the Netherlands; 4 Department of Intensive Care, Ikazia hospital, Rotterdam, the Netherlands

**Keywords:** Single-occupancy rooms, Nurses’ satisfaction, Staff experience, Hospital design

## Abstract

**Background:**

To improve patients’ privacy, comfort and infection control, newly built hospitals increasingly offer 100% single-occupancy patient rooms. Our study examines how nurses perceived the transition from a hospital with multi-bedded patient rooms to one with solely single-occupancy patient rooms designed according to principles of a healing environment.

**Methods:**

In a single-centre, before-after survey study, nurses completed a questionnaire of 21 items in three domains: perceived patient safety and monitoring, nurses’ working conditions and patient environment. Before-measurements (*n* = 217) were compared with two after-measurements in the new hospital, respectively after one (*n* = 483) and two years (*n* = 191).

**Results:**

Nurses considered the single rooms in the new hospital worse for visibility and monitoring but this had improved somewhat after two years. In either setting, the majority perceived working conditions (walking distances and designated rest area) as unfavourable. The patient environment in the new hospital was generally perceived as much better than in the former hospital.

**Conclusion:**

The transition to solely single-occupancy patient rooms was largely considered positive by nurses in terms of patient environment. However, monitoring of patients and working conditions remain a concern. When designing new hospitals, attention should be paid to optimal working conditions for nurses. To improve monitoring of patients, we recommend the use of remote-sensoring.

**Supplementary Information:**

The online version contains supplementary material available at 10.1186/s12912-024-01758-7.

## Background

Worldwide, the proportion of single-occupancy patient rooms (SPRs) in newly built hospitals is growing [1, 2]. Benefits of this trend include improved patient privacy and wellbeing [3], facilitation of family presence [4] and improved communication between patient and health care workers [5, 6]. Furthermore, infection control may be more effective in hospitals with SPRs [7, 8]. As described by Maben and colleagues [1], possible disadvantages of SPRs include reduced social interaction and risks of patient isolation. Moreover, less surveillance of medical and nursing staff and lack of social control by fellow patients may increase the falls incidence [9]. For hospital staff, an improvement in the quality of the work environment is described, with more time available for patients than in a setting with multi-bedded rooms and more personalized care with fewer interruptions by colleagues [1, 2]. However, team performance may decrease in a hospital with SPRs, as there are fewer interactions between staff, leading to more challenging interpersonal relationships and communication [2, 10].

In May 2018, a large University Medical Centre in the Netherlands relocated to a newly built adult hospital with 100% single-occupancy patient accommodations. The transition to SPRs primarily aimed to reduce the number of hospital-acquired infections, to increase occupancy rates as well as to optimize the healing circumstances for patients. The new hospital was designed in accordance with infection control guidance and evidence based design interventions originating from healing environment principles. Factors such as privacy, visual and acoustic comfort, ample daylight, presence of art and (views of) nature and patient control over room temperature and lighting facilitate positive health outcomes [11]. In addition nurses’ work processes were adapted to the new ward environment and standardized over the general wards. New roles were introduced on the ward (e.g. pharmacy assistant and facility care worker) and new IT- and support systems came in use at relocation.

In this study we collected perspectives of nurses on general wards and intensive care units before and after the transition to the new hospital. Our research question was whether the new environment, in conjunction with the other changes that took place at relocation, indeed offers more benefits for the nursing staff and for the care provided to their patients.

## Methods

### Design

We used an uncontrolled before and after design for this single-centre survey study. ‘Before’-data were collected from November 2017 through January 2018 (former hospital), followed by two periods of ‘after’-data collection (new hospital): from November 2018 through January 2019 and from February 2020 through August 2020. The second ‘after’-data collection covered a longer period because of Covid-19 restrictions in our hospital.

### Setting

The general wards in the former hospital mainly contained four-bedded and two-bedded patient rooms, but also one or two SPRs per ward. The wards had a typical ‘racetrack’ configuration and held around 32 beds. The new wards also have around 24–32 beds, that are mostly clustered in groups of eight rooms, with a decentralized touch-down nursing station. An on-stage/off-stage distinction was intended in the design. The characteristics of the patient rooms in the former and new hospital are shown in Table 1. The patient rooms on the intensive care units in the former hospital were all single patient rooms with camera surveillance, which was no longer allowed in the new hospital for privacy reasons. A total of approximately 2,500 nurses were employed in the former and the new hospital.

**Table 1:** Characteristics of patient rooms in former and new hospital in the general wards

	Former hospital	New hospital
**Number of patients in room**	One, two or four	One
**Bathroom and toilet**	In hallway (for max.12 patients)	Ensuite bathroom
**Room lighting control**	Only bed light controlled by patient	All lighting controlled by patient (by patient-tablet)
**Temperature control in room**	Not possible	Controlled to some extent by patient (by patient tablet)
**Room doors**		
- **Standard**	Most with small window	Without window
- **Pressurized rooms**	With windows in both doors	With window in both doors
**Room interior design**	Standard, privacy curtain around the beds	Wooden door and facade, windows can be opened, soothing colors, orientation light under vertical bedhead panel; no privacy curtain
**Sofa bed for rooming-in**	No	Yes
**Ceiling hoist**	No	Yes
**Nurse call system**		
- **Patient (activating)**	Button near bed and cord in bathroom	Button near bed and alarm on wrist-band
- **Nurse (receiver)**	Light above door and alarm tone	Alarm on nurse’s portable device

### Sample

Before-measurements were carried out over 52 days in the former hospital, followed by two periods of after-measurements in the new hospital (period one, 75 days, after one year and period two, 198 days, after two years). The second after-measurement served to assess to what extent habituation had played a role in hospital staff’s opinions of the new hospital. The study population (three convenience samples) consisted of hospital staff– mainly nursing staff– on twelve wards in the former hospital and twenty-five wards in the new hospital. The difference in the number of wards can be explained by the transition from largely homogeneous departments in the old hospital to care centers in the new hospital. The wards included surgical wards, medical wards, high dependency units and intensive care units.

### Instruments used

A ‘ward environment’ questionnaire was administered, which was a modification of one developed by Maben [1]. First, items which were considered more appropriate to patients were removed. Examples are items about patient’s privacy and sanitary facilities. Items that did not concern ward design were also removed e.g. availability of computers. We did add two items about environmental noise during day and night shifts. This resulted in 21 statements concerning three topics: “perceived patient safety, monitoring opportunities and ease of collaboration”, “nurse working conditions” and “patient environment”. Answer options on a five-point Likert-scale ranged from ‘totally disagree’ to ‘totally agree’, with a middle category ‘not disagree, not agree’. In addition, the option ‘not applicable’ was provided. Furthermore, respondents’ age, sex, profession and years of work experience were collected as demographic data.

### Data collection methods

The collection of data was done by students, who visited the patient wards and units with portable computers (tablets). We refrained from collecting data on the items concerning ‘patient environment’ in the second period of the new hospital, because the first ‘after’-data collection had shown an obviously improved agreement with these statements, and also to limit the burden on the nursing staff. The collected data were stored on a secure server.

### Data analysis

For analysis of the descriptive data, we used IBM Statistical Package of the Social Sciences for Windows (SPSS), version 19 (IBM Corp., Armonk, N.Y., USA).

### Ethical considerations

The study was approved by the Medical Ethics Review Board of our hospital (MEC 2017 − 1103).

## Results

Two hundred and seventeen participants were involved in the before-measurement versus 483 in the first after-measurement and 191 in the second after-measurement. Participant characteristics are shown in Table 2. Due to outflow and inflow of employees in the hospital during this period of time (2017–2020) and different response rates, the three participant groups were neither identical in size nor in years of working in hospital. Of a total of 891 participants (total of pre- and post-measurements), 648 (72.7%) completed the questionnaire non-anonymously. Of these, eighty-four (13.0%) completed the questionnaires twice and two participants completed them three times.

**Table 2:** Characteristics of respondents

	Former hospital *N* = 217	New hospital period 1 *N* = 483	New hospital period 2 *N* = 191
**Age, median** (interquartile range)	28 (24–46)	29 (23–41)	29 (24–41)
**Sex: female** (%)	172 (79.3)	409 (84.7)	140 (73.3)^†^
**Profession**			
Nurse (%)	139 (64.1)	377 (78.0)	130 (68.1)
Student-nurse (%)	40 (18.4)	99 (20.5)	39 (20.4)
Medical student (%)	4 (1.8)	3 (0.6)	- (-)
Clinical/doctor’s assistant (%)	10 (4.6)	4 (0.9)	3 (1.6)
Unknown (%)	24 (11.1)	- (-)	19 (9.9)
**Working at**			
General wards, n (%)	180 (82.9)	420 (87.0)	148 (77.5)
Intensive care unit, n (%)	37 (17.1)	63 (13.0)	43 (22.5)
**Years working in hospital, median** (interquartile range)	7 (2–17) §	4 (1–13)	6 (2–11) ¶

†) 16 missing values (8%); §) 28 missing values (13%); ¶) 182 missing values (95%)

### Perceived patient safety, monitoring opportunities and ease of collaboration

**Fig. 1:**
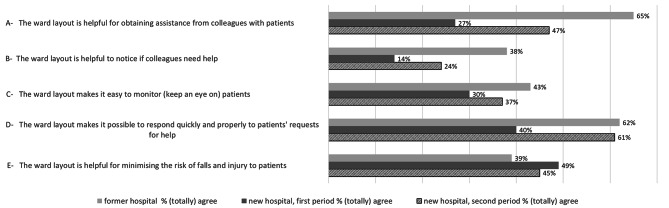
Perceived patient safety, monitoring opportunities and ease of collaboration Proportions of participants who (totally) agreed with the statements concerning perceived patient safety, monitoring opportunities and ease of collaboration, for former and new hospital (first and second data collection periods)

Figure 1 shows the proportions of participants who (totally) agreed with the statements concerning patient safety, monitoring opportunities and ease of collaboration, broken down for the former and new hospital (first and second periods). At baseline 65% of the participants in the old hospital considered the ward layout helpful for obtaining assistance from colleagues (item A), whereas this declined to 27% in the first after-assessment but improved again in the second after-assessment (47%). Overall agreement was lower in the first period of the new hospital than in the former hospital, although this increased in the second period of the new hospital for items A to D. Concerning the risk of falls and injury to patients (item E), slightly more participants considered the ward layout helpful in the new hospital (respectively 49% and 45% agreed) compared to 39% in the former hospital. Overall, patient safety, monitoring opportunities and ease of collaboration were considered to have worsened in the first period in the new hospital, with improved appreciation a year later (see additional file 1).

### Nurses’ working conditions

**Fig. 2:**
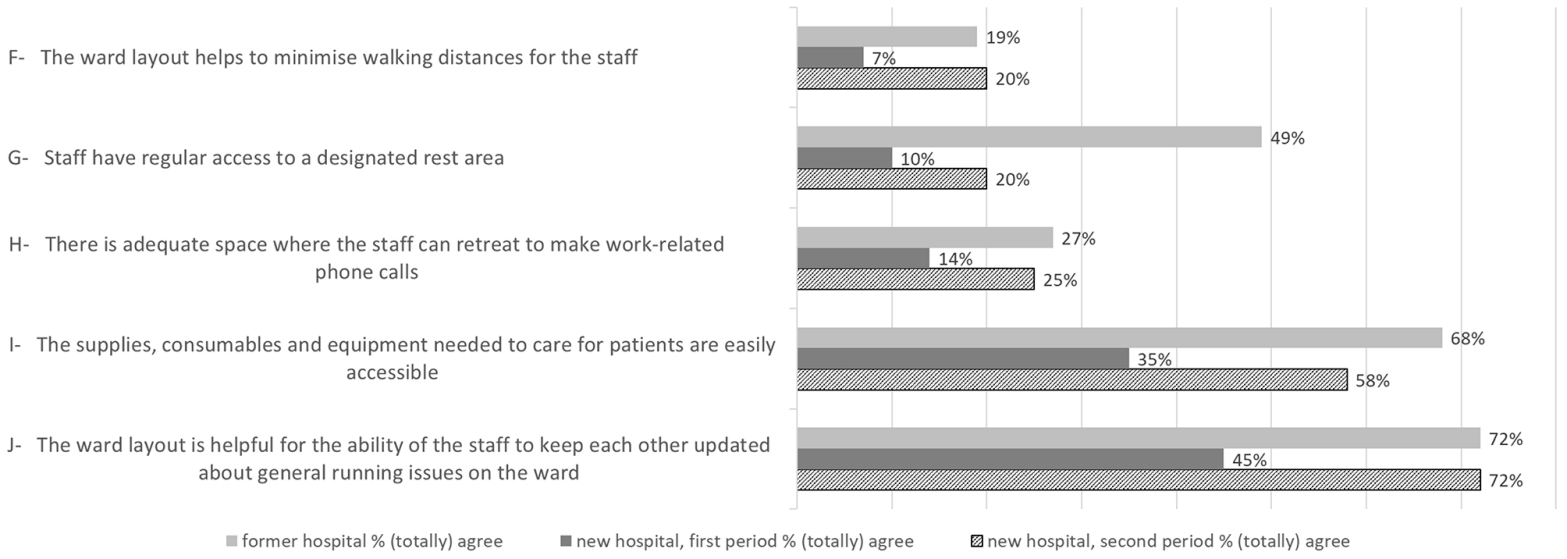
Nurse working conditions Proportions of participants who (totally) agreed with the statements concerning nurses’ working conditions, for former and new hospital (first and second data collection periods)

Figure 2 provides the proportions of participants who (totally) agreed with the statements concerning nurses’ working conditions, for the former and new hospital (first and second periods). With regard to all the items, we see a decrease in appreciation of the working conditions in the first period of the new hospital compared to the former hospital. In the second period, appreciation improved again for the items concerning helpfulness to minimize walking distances (item F), accessibility of supplies, consumables and equipment needed for care (item I) and helpfulness for the staff to keep each other updated about general running issues on the ward (item J). In general, the nurses’ working conditions were considered to have worsened in the first period in the new hospital, with an improvement a year later. In additional file 2, data are shown concerning responses on nurses’ working conditions.

### Patient environment

**Fig. 3:**
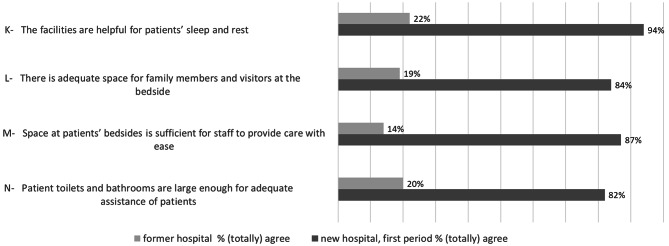
Patient environment Proportions of participants who (totally) agreed with the statements concerning patient environment, for former and new hospital (first period)

Figure 3 shows that in the former hospital the patient environment was rarely perceived as positive: 14 to 22% agreed with the items concerning helpfulness of the facilities for patients’ sleep and rest (item K), the adequateness of space at the bedside for family and visitors (item L) and for staff (item M) and the item concerning the size of the patient toilets and bathrooms with regard to adequate assistance (item N). Whereas in the new hospital the vast majority of the participants was positive about the patient environment (82 to 94% agreed or totally agreed with these items). All data concerning responses on patient environment are shown in additional file 3.

## Discussion

In this uncontrolled before-after survey study, we investigated how nurses perceived the benefits of the transition from mainly multi-bedded patient rooms to 100% single-occupancy rooms in a newly built hospital. We did two after-measurements after the transition to the new hospital. Patient safety, patient monitoring opportunities, ease of collaboration and nurse working conditions were perceived worse in the new hospital than in the former hospital with mainly two- and four-bedded rooms. However, the questionnaire results indicate improved appreciation more than one year later in the new hospital. This can perhaps be ascribed to habituation to the new situation. This situation not only consists of the new built environment but does include adjustment to new routines, equipment and team composition [12]. The different compositions of the three study populations may also have played a role: we may reasonably assume that the second after-measurement included more nurses who had not worked in the former hospital than in the first after-measurement. Furthermore, several adjustments had been made in the new hospital, which may have contributed to this improvement. For example, to reduce walking distances for the staff and to shorten the response time to patients’ request for help, work processes were reorganized by means of creating several sub-teams within a ward.

Both in the former and the new hospital, most nurses were not positive about certain aspects of the ward layout, such as noticing whether a colleague needed help. It would be interesting to explore if technology could help to improve this. For instance, using hand-free ways to always be in touch instead of the currently used mobile phones. As another example, in the current setting with only single patient rooms, nurses of general wards found monitoring of patients difficult because for instance of closed doors without windows. Even in the second after-measurement, less than half of the respondents considered the ward layout helpful in monitoring patients. Better patient monitoring is needed, especially for high risk patients: older, vulnerable or delirious patients. To improve patient safety and also nurses’ sense of being able to work safely, remote patient monitoring, based on non-invasive and wearable sensors is likely to be used more often in the future [13]. In this hospital, a pilot study has started on the usefulness of such remote monitoring.

There is also room for improvement regarding the ease of noticing that a colleague needs help or can assist you. This has also consequences for the learning climate for student nurses in our teaching hospital, which deserves attention and has been noted elsewhere [14]. If nurses have difficulty finding each other, it will be less easy for them to get assistance [15, 16].

Considering the high workload of nurses, excellent working conditions are crucial. We think it is important to provide spaces with sufficient privacy for nurses to take a break, make phone calls or supervise students. The new hospital did provide designated rest areas with sufficient privacy, but they were not used as intended. Infection control policy discouraged eating and drinking in the on-stage area; the off-stage area was perceived as being too distant or inappropriate, as the team room doubled as meeting room or was considered to be too small. In the former wards the kitchen cum restroom was situated at the heart of the unit, but this mixed use was not in accordance with infection control policy. Infection control was, however, an important driver in designing the new hospital. Van der Schoor and colleagues from our center studied the effect of the transition to solely SPRs on infection control. They found a significant reduction of environmental contamination with highly resistant microorganisms (HRMO) after the relocation [17]. A significant decrease in acquisition of Extended-spectrum beta-lactamase-producing Enterobacterales (a well-known cause of healthcare-associated infections) after the relocation could not be shown, given the low prevalence before and after the relocation, but the researchers noted a significant decrease in the number of intra-hospital patient transfers [8].

With regard to the patient environment (e.g. bedside space, ensuite bathroom), participating nurses appreciated the single patient rooms much more than the multi-bedded rooms. The transition to SPRs is considered largely positive for patients, but risks of social isolation and hampered patient mobilization, as well as perceived falls risks remain a concern, as pointed out by Maben and colleagues [1, 2]. In a study by our group, the incidence of falls was 1.39 per 1,000 patient days in the former hospital and 1.38 per 1,000 patient days in the new hospital (*p* = 0.924) [18]. Consequently, concerns that the patient fall rate would increase after the relocation to solely single-occupancy patient rooms were not confirmed.

We have surveyed patient satisfaction with facilities before and after the relocation, using a modified version of the questionnaire used by Maben et al. focusing on patients’ perspectives of the environment [1]. Patients were especially satisfied about the improved privacy, although approximately a quarter sometimes missed the company of fellow patients (reporting of this before-after survey study in our center will be forthcoming).

### Strengths and limitations

A strength of this study is that we performed two after-measurements following the transition to the new hospital, to capture any habituation to the new hospital. Several limitations of the study need to be addressed. First there was no control hospital involved, which limits the strength of the conclusions, as it is clear that the relocation involved more changes for nurses to get accustomed to than just the built environment. Second, the sample sizes across the three measurements varied considerably which might have impacted the results although in what way is hard to estimate. The impact of the proportion of ICU nurses is a factor that might have influenced our findings, as ICU-beds in the old hospital were already provided in SPRs. Third, because of restrictions due to Covid-19 during the second after-measurement, this measurement was smaller, more spread out in time and less structured than the other measurements (and some wards had been repurposed for Covid-19-care and other wards relocated to a recommissioned building, with a traditional ward configuration). Fourth, our study used convenience samples (not matched) and staff turnover meant that many final respondents had not worked in the original hospital, which hampered comparison of the three measurement periods. Fifth, with hindsight it is unfortunate that the questions about e.g. availability of computers were removed from the survey, as the ward environment with decentralized nursing stations also offered a different model of IT-support for nurses’ work processes. Finally, the study did not include an in-depth analysis of factors affecting nurses’ workflow, the results of which could have been of added value.

## Conclusion

Nurses considered the 100% single-occupancy ward configuration largely positive in terms of patient (and ward) environment. Patient safety, patient monitoring opportunities, ease of collaboration and nurse working conditions were perceived as worse in the new hospital, which perception was less outspoken one year later in the new hospital. This may be due to reorganized work processes and habituation to the new situation. Several design elements in the new hospital were not conducive to optimized work processes for the nursing staff. Resolving these issues may have a positive effect on the quality of care provided, but confirmation requires additional data. Because monitoring of patients remained a concern, the use of remote sensoring of vital signs is recommended. Lastly, when designing new hospitals, attention should be paid to optimal working conditions for nurses, ways to easily locate colleagues, and providing adequate rest facilities.

### Relevance to clinical practice

This study emphasizes the importance of adequate patient monitoring, which is challenging in a setting with 100% single-occupancy rooms. Remote monitoring using sensoring devices could be one way to improve patient safety. Furthermore, it is important to provide spaces for nurses where they can work, teach and relax in privacy. These off-stages spaces should be provided in close proximity of the patient rooms.

### Electronic supplementary material

Below is the link to the electronic supplementary material.

Supplementary Material 1

Supplementary Material 2

Supplementary Material 3

Supplementary Material 4 Supplementary Material 4

Supplementary Material 5 Supplementary Material 5

## Data Availability

The datasets used and analysed during the current study are available from the corresponding author on reasonable request.

## Abbreviations

ICU: intensive care unit
IT: information technology
SPRs: single-occupancy patient rooms
