# Supplementary material for: Nurses’ perceptions of the transition to 100% single-occupancy patient rooms in a university hospital in the Netherlands: an uncontrolled before and after study
Source: BMC Nurs. 2024 Feb 8;23:106. doi: 10.1186/s12912-024-01758-7 (PMC10851588; doi:10.1186/s12912-024-01758-7)
Supplement: Supplementary file 1 — Supplementary Material 1 [file 12912_2024_1758_MOESM1_ESM.docx]

**Additional file 1:** **Perceived patient safety, monitoring opportunities and ease of collaboration**

| **Question** | **Former hospital, n (%)** | | | **New hospital, episode 1, n (%)** | | | **New hospital, episode 2, n (%)** | | |
| --- | --- | --- | --- | --- | --- | --- | --- | --- | --- |
|  | **(Totally) disagree** | **Not disagree, not agree** | **(Totally) agree** | **(Totally) disagree** | **Not disagree, not agree** | **(Totally) agree** | **(Totally) disagree** | **Not disagree, not agree** | **(Totally) agree** |
| The ward layout is helpful for obtaining assistance from colleagues with patients | 35 (16.3) | 40 (18.6) | 140 (65.1) | 256 (53.8) | 91 (19.1) | 129 (27.1) | 56 (29.6) | 44 (23.3) | 89 (47.1) |
| The ward layout is helpful to notice if colleagues need help | 82 (38.1) | 52 (24.2) | 81 (37.7) | 329 (69.1) | 82 (17.2) | 65 (13.7) | 101 (52.9) | 44 (23.0) | 46 (24.1) |
| The ward layout makes it easy to monitor (keep an eye on) patients | 80 (37.6) | 41 (19.2) | 92 (43.2) | 242 (51.0) | 90 (18.9) | 143 (30.1) | 76 (40.2) | 43 (22.8) | 70 (37.0) |
| The ward layout makes it possible to respond quickly and properly to patients' requests for help | 33 (15.5) | 47 (22.1) | 133 (62.4) | 150 (31.4) | 138 (28.9) | 189 (39.6) | 33 (17.4) | 40 (21.2) | 116 (61.4) |
| The ward layout is helpful for preventing and controlling hospital-acquired infections | 45 (20.9) | 39 (18.1) | 131 (61.0) | 45 (9.5) | 90 (19.0) | 338 (71.5) | - | - | - |
| The ward layout is helpful for minimising the risk of falls and injury to patients | 71 (34.1) | 57 (27.4) | 80 (38.5) | 123 (26.3) | 115 (24.6) | 230 (49.1) | 56 (29.8) | 47 (25.0) | 85 (45.2) |
| The answer possibility ‘N.A.’ is handled as missing value. | | | | | | | | | |
