# Supplementary material for: Nurses’ perceptions of the transition to 100% single-occupancy patient rooms in a university hospital in the Netherlands: an uncontrolled before and after study
Source: BMC Nurs. 2024 Feb 8;23:106. doi: 10.1186/s12912-024-01758-7 (PMC10851588; doi:10.1186/s12912-024-01758-7)
Supplement: Supplementary file 2 — Supplementary Material 2 [file 12912_2024_1758_MOESM2_ESM.docx]

**Additional file 2: Nurses’ working conditions**

| **Question** | **Former hospital, n (%)** | | | **New hospital, episode 1, n (%)** | | | **New hospital, episode 2, n (%)** | | |
| --- | --- | --- | --- | --- | --- | --- | --- | --- | --- |
|  | **(Totally) disagree** | **Not disagree, not agree** | **(Totally) agree** | **(Totally) disagree** | **Not disagree, not agree** | **(Totally) agree** | **(Totally) disagree** | **Not disagree, not agree** | **(Totally) agree** |
| The ward layout helps to minimise walking distances for the staff | 107 (49.8) | 67 (31.2) | 41 (19.0) | 393 (82.0) | 54 (11.3) | 32 (6.7) | 112 (58.6) | 40 (20.9) | 39 (20.4) |
| Staff have regular access to a designated rest area | 70 (32.7) | 39 (18.2) | 105 (49.1) | 367 (76.0) | 66 (13.7) | 50 (10.3) | 125 (65.4) | 28 (14.6) | 38 (20.0) |
| There is adequate space where the staff can retreat to make work-related phone calls | 108 (50.2) | 50 (23.3) | 57 (26.5) | 336 (71.0) | 69 (14.6) | 68 (14.4) | 110 (57.6) | 34 (17.8) | 47 (24.6) |
| The supplies, consumables and equipment needed to care for patients are easily accessible | 34 (16.0) | 33 (15.6) | 145 (68.4) | 201 (42.2) | 109 (22.9) | 166 (34.9) | 38 (20.3) | 40 (21.4) | 109 (58.3) |
| The ward layout is helpful for the ability of the staff to keep each other updated about general running issues on the ward | 20 (9.3) | 40 (18.6) | 155 (72.1) | 147 (30.6) | 116 (24.2) | 217 (45.2) | 20 (10.5) | 34 (17.9) | 136 (71.6) |
| The number of clinical hand wash basins supports good hand hygiene | 33 (15.4) | 16 (7.4) | 166 (77.2) | 46 (9.5) | 20 (4.2) | 417 (86.3) | - | - | - |
| The location of clinical hand wash basins supports good hand hygiene | 54 (25.0) | 26 (12.0) | 136 (63.0) | 90 (18.7) | 48 (9.9) | 344 (71.4) | - | - | - |
| There is adequate light for the staff during the day | 34 (15.7) | 16 (7.4) | 167 (76.9) | 45 (9.3) | 35 (7.3) | 401 (83.4) | - | - | - |
| There is adequate light for the staff during the night | 28 (16.7) | 21 (12.5) | 119 (70.8) | 34 (9.3) | 37 (10.1) | 296 (80.6) | 9 (5.6) | 11 (6.8) | 141 (87.6) |
| Ambient sounds such as equipment, squeaking doors, telephones, loud voices, clogs etc disturb me during the day | 74 (34.3) | 58 (26.9) | 84 (38.9) | 216 (45.3) | 102 (21.4) | 159 (33.3) | - | - | - |
| Ambient sounds such as equipment, squeaking doors, telephones, loud voices, clogs etc disturb me during the night | 48 (28.6) | 44 (26.2) | 76 (45.2) | 194 (51.6) | 72 (19.1) | 110 (29.3) | - | - | - |
| The answer possibility ‘N.A.’ is handled as missing value. | | | | | | | | | |
