# Supplementary material for: Nurses’ perceptions of the transition to 100% single-occupancy patient rooms in a university hospital in the Netherlands: an uncontrolled before and after study
Source: BMC Nurs. 2024 Feb 8;23:106. doi: 10.1186/s12912-024-01758-7 (PMC10851588; doi:10.1186/s12912-024-01758-7)
Supplement: Supplementary file 3 — Supplementary Material 3 [file 12912_2024_1758_MOESM3_ESM.docx]

| **Question** | **Former hospital , n (%)** | | | **New hospital , episode 1, n (%)** | | | **New hospital , episode 2, n (%)** | | |
| --- | --- | --- | --- | --- | --- | --- | --- | --- | --- |
|  | **(Totally) disagree** | **Not disagree, not agree** | **(Totally) agree** | **(Totally) disagree** | **Not disagree, not agree** | **(Totally) agree** | **(Totally) disagree** | **Not disagree, not agree** | **(Totally) agree** |
| Patient toilets and bathrooms are large enough for adequate assistance of patients | 122 (61.9) | 35 (17.8) | 40 (20.3) | 31 (6.8) | 52 (11.4) | 374 (81.8) | - | - | - |
| Space at patients’ bedsides is sufficient for staff to provide care with ease | 153 (71.8) | 30 (14.1) | 30 (14.1) | 16 (3.3) | 49 (10.2) | 416 (86.5) | - | - | - |
| There is adequate space for family members and visitors at the bedside | 137 (64.9) | 34 (16.1) | 40 (19.0) | 28 (5.9) | 49 (10.4) | 394 (83.7) | **-** | **-** | **-** |
| The facilities are helpful for patients’ sleep and rest | 116 (54.2) | 52 (24.3) | 46 (21.5) | 6 (1.3) | 24 (5.0) | 448 (93.7) | **-** | **-** | **-** |
| The answer possibility ‘N.A.’ is handled as missing value. | | | | | | | | | |

**Additional file 3: Patient environment**
