# Supplementary figures and images for: Nurses’ perceptions of the transition to 100% single-occupancy patient rooms in a university hospital in the Netherlands: an uncontrolled before and after study
Source: BMC Nurs. 2024 Feb 8;23:106. doi: 10.1186/s12912-024-01758-7 (PMC10851588; doi:10.1186/s12912-024-01758-7)

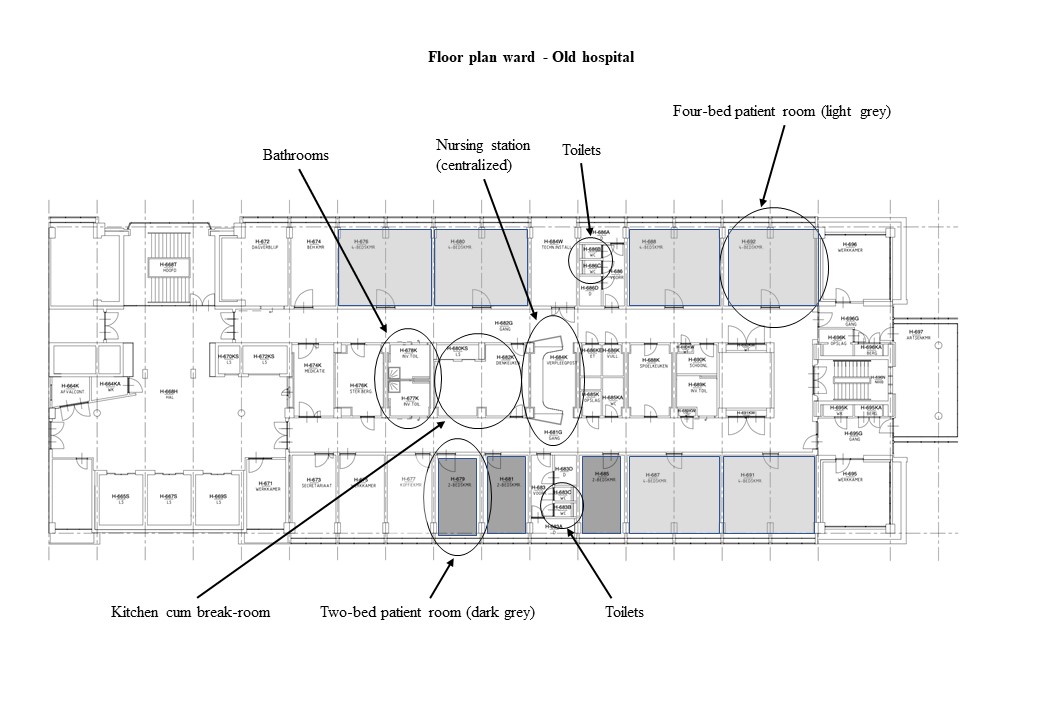

Supplement: Supplementary file 4 — Supplementary Material 4 [file 12912_2024_1758_MOESM4_ESM.jpg]

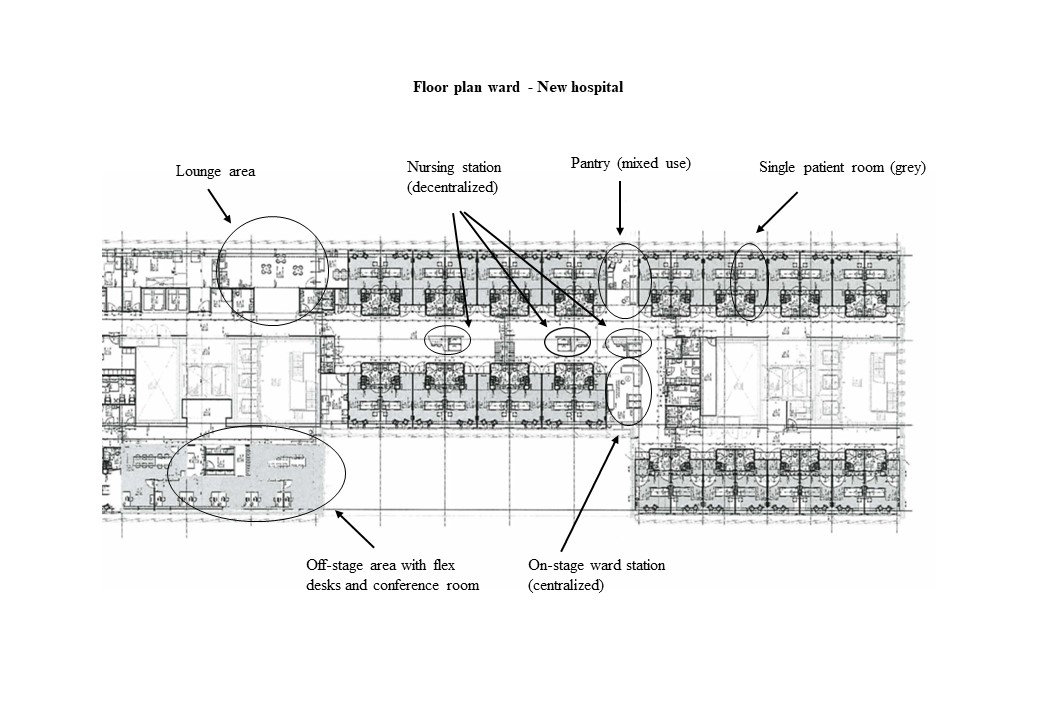

Supplement: Supplementary file 5 — Supplementary Material 5 [file 12912_2024_1758_MOESM5_ESM.jpg]
